# Genetic Copy Number Variation and General Cognitive Ability

**DOI:** 10.1371/journal.pone.0037385

**Published:** 2012-12-26

**Authors:** Andrew K. MacLeod, Gail Davies, Antony Payton, Albert Tenesa, Sarah E. Harris, David Liewald, Xiayi Ke, Michelle Luciano, Lorna M. Lopez, Alan J. Gow, Janie Corley, Paul Redmond, Geraldine McNeill, Andrew Pickles, William Ollier, Michael Horan, John M. Starr, Neil Pendleton, Pippa A. Thomson, David J. Porteous, Ian J. Deary

**Affiliations:** 1 Centre for Cognitive Ageing and Cognitive Epidemiology, University of Edinburgh, Edinburgh, United Kingdom; 2 Medical Genetics Section, Centre for Molecular Medicine, Institute of Genetics and Molecular Medicine, University of Edinburgh, Edinburgh, United Kingdom; 3 Department of Psychology, University of Edinburgh, Edinburgh, United Kingdom; 4 Centre for Integrated Genomic Medical Research, University of Manchester, Manchester, United Kingdom; 5 The Roslin Institute, Royal (Dick) School of Veterinary Studies, University of Edinburgh, Edinburgh, United Kingdom; 6 Medical Research Council Human Genetics Unit, Institute of Genetics and Molecular Medicine, University of Edinburgh, Edinburgh, United Kingdom; 7 Medical Research Council Centre of Epidemiology for Child Health, University College London Institute of Child Health, London, United Kingdom; 8 Institute of Applied Health Sciences, University of Aberdeen, Aberdeen, United Kingdom; 9 School of Epidemiology and Health Science, Department of Medicine, University of Manchester, Manchester, United Kingdom; 10 School of Community-Based Medicine, Neurodegeneration Research Group, University of Manchester, Manchester, United Kingdom; 11 Geriatric Medicine Unit, University of Edinburgh, Edinburgh, United Kingdom; Oklahoma Medical Research Foundation, United States of America

## Abstract

Differences in genomic structure between individuals are ubiquitous features of human genetic variation. Specific copy number variants (CNVs) have been associated with susceptibility to numerous complex psychiatric disorders, including attention-deficit-hyperactivity disorder, autism-spectrum disorders and schizophrenia. These disorders often display co-morbidity with low intelligence. Rare chromosomal deletions and duplications are associated with these disorders, so it has been suggested that these deletions or duplications may be associated with differences in intelligence. Here we investigate associations between large (≥500kb), rare (<1% population frequency) CNVs and both fluid and crystallized intelligence in community-dwelling older people. We observe no significant associations between intelligence and total CNV load. Examining individual CNV regions previously implicated in neuropsychological disorders, we find suggestive evidence that CNV regions around *SHANK3* are associated with fluid intelligence as derived from a battery of cognitive tests. This is the first study to examine the effects of rare CNVs as called by multiple algorithms on cognition in a large non-clinical sample, and finds no effects of such variants on general cognitive ability.

## Introduction

Among humans, individual differences in measured intelligence are associated with important life outcomes, including long-term health and wellbeing [1], [2]. General cognitive ability (usually denoted *g*) is a quantitative trait, and is assessed using cognitive ability tests. Empirical evidence for *g* was first described by Spearman [3] who found that diverse mental capabilities tended to show positive covariation. The general intelligence factor, *g*, accounts for about 50% of the total variance when a number of diverse mental tests are administered to population samples [4]. The *g* factors derived from very different cognitive test batteries rank people almost identically [5], and intelligence differences are highly stable across the human lifecourse [6]. Two major facets of intelligence are crystallized (*g_c_*) and fluid (*g_f_*) intelligence. Crystallized-type intelligence is characterised by a relative lack of decline with age [7], and is typically assessed using tests of acquired knowledge, most often vocabulary. Types of cognitive ability that are termed ‘fluid intelligence’ tend to decline with age from young or middle adulthood [7], and are assessed using unfamiliar, sometimes abstract materials, and involve on-the-spot thinking, often under time pressure, and rely relatively little on prior knowledge. Intelligence is substantially heritable [8], [9] and, although variants in a number of candidate genes have shown significant associations, few have replicated [10], [11]. This is true for many complex traits: even in the most highly heritable, such as height, known variants account for only a small proportion of the observed heritability [12]. Several hypotheses have been proposed as to where this “missing heritability” resides [13], [14]. These include common variants of small effect [15], rare variants with large effects [16], epistatic interactions [17], epigenetic factors [18] as well as other forms of genetic variation beyond single nucleotide polymorphisms (SNPs). One example of this last factor is structural genetic variation, which includes copy number variation.

Copy number variants (CNVs) are defined as segments of DNA longer than 1kb present in variable numbers of copies across individuals within a population sample [19]. CNVs are defined relative to a normal copy number of two; hence, segments that are present in more than two copies within an individual are classed as duplications, and fewer than two are classed as deletions. CNVs are observed ubiquitously throughout the genomes of humans [20] and other organisms [21]. Older SNP genotyping arrays missed some of this variation, but recent high density arrays include multiple non-polymorphic markers in regions of known structural variation, allowing more reliable detection of CNVs.

The Database of Genomic Variants [22] reports over 60,000 CNVs at more than 15,000 loci from 42 reported studies, that collectively cover more than a third of the human genome. Whereas 1kb is typically taken as the minimum length for a CNV, the largest can span several megabases, and can potentially disrupt multiple genes and/or regulatory regions, each of which may have an effect on gene expression and phenotype [23]. Initial studies of CNV prevalence [20] suggested extensive numbers of smaller CNVs across populations. There is still some debate regarding the best method to detect CNVs from SNP data [24], [25]. Different methods show marked variation in the number and extent of CNVs detected from the same samples, and the reliability of calling shorter CNVs is especially questionable. However, calls for longer CNVs are more consistent between methods [26], are more likely to represent true variants, and thus have the potential for more robust replication. Therefore, the current study examines the effect of longer, rare CNVs on human intelligence differences.

Specific CNVs have been associated with susceptibility to illnesses including HIV-1 infection [27], autoimmune disorders [28]–[31], and cancer [32]; nervous system disorders [33] such as Alzheimer's Disease [34], Parkinson's Disease [35], [36], epilepsy [37]; and psychiatric disorders, including schizophrenia [38], [39], mental retardation [40]–[42], autism [43]–[46] and major depressive disorder [47]. However, as most detected CNVs are relatively rare within discovery cohorts [48], association tests for individual CNVs will be of limited power to detect significant variants. An alternative approach is to test jointly for the effect of multiple rare CNVs on disease status, by comparing the overall CNV load between cases and controls. The overall CNV load can be measured as the total number of rare CNVs carried, the total length of these CNVs, or the total number of genes they disrupt. The method of examining overall CNV load has been applied to a number of psychiatric and neurological disorders [49]–[51]. Here, we apply it to variation in human intelligence, treated as a quantitative trait, measured in community-dwelling older people.

A report by Yeo *et al.* [52] identified a significant association between the extent of rare genetic deletions and Full-Scale Intelligence Quotient (FSIQ), derived from the Wechsler abbreviated scale of intelligence [53], in a very small clinical sample of patients undergoing treatment for alcoholism. The authors acknowledged that there have also been findings of CNV differences between controls and disorders that involve cognitive deficits. Williams *et al.* [54] examined the effect of rare CNVs on risk of Attention-Deficit Hyperactivity Disorder (ADHD), finding significant differences in numbers of CNVs ≥500 kb between cases and controls for total CNV burden. Patients with psychiatric disorders such as schizophrenia and ADHD often display cognitive deficits [55], suggesting that the burden associated with rare CNVs may also have an effect on intelligence itself. Here we present analyses of the effect of large, rare CNVs on measured intelligence in cohorts of relatively healthy individuals with a total sample size of more than 3,000 older people.

## Methods

### Ethics Statement

Ethical approval for the Lothian Birth Cohort 1921 study was obtained from the Lothian Research Ethics Committee. Ethical approval for the Lothian Birth Cohort 1936 study was obtained from Scotland's Multicentre Research Ethics Committee and the Lothian Research Ethics Committee. Ethical approval for the Aberdeen Birth Cohort 1936 study was obtained from the Grampian Research Ethics Committee. Ethical approval for Manchester and Newcastle Longitudinal Studies of Cognitive Ageing Cohorts study was obtained from the University of Manchester. Written consent was received from all participants for their information to be stored in the relevant university database and used for research.

### Cohort Descriptions

#### Lothian Birth Cohort 1921

The Lothian Birth Cohort 1921 (LBC1921) is a longitudinal study of cognitive ageing conducted at the University of Edinburgh. Individuals in the LBC1921 were born in 1921 and were recruited and tested in old age, as described elsewhere [6], [56]. In total, 550 individuals (234 male, 316 female) were tested at mean age 79.1 years (SD = 0.6). Participants were tested individually, and completed a battery of cognitive tests including: The Moray House Test No. 12 (MHT) [57], Raven's Standard Progressive Matrices [58], Verbal Fluency [59], and Logical Memory [60]. Participants also completed the National Adult Reading Test (NART) [61].

#### Lothian Birth Cohort 1936

The Lothian Birth Cohort 1936 (LBC1936) is a longitudinal study of cognitive ageing conducted at the University of Edinburgh. Individuals in the LBC1936 were born in 1936 and were recruited and tested in old age, as described elsewhere [62]. In total, 1091 individuals (548 male, 543 female) were tested at a mean age of 69.5 years (SD  = 0.8). Participants were tested individually on a large battery of cognitive tests [62] including the MHT, and the following six tests from the Wechsler Adult Intelligence Scale-III^UK^ (WAIS-III^UK^ [60]): Digit Symbol Coding, Block Design, Matrix Reasoning, Digit Span Backwards, Symbol Search, and Letter-number Sequencing. Participants also completed the NART [61].

#### Aberdeen Birth Cohort 1936

The Aberdeen Birth Cohort 1936 (ABC1936) is a longitudinal study of cognitive ageing. Individuals in the ABC1936 were born in 1936 and were recruited and tested in old age as described elsewhere [6], [56]. In total, 498 individuals (243 men, 255 women) were tested at mean age 64.6 years (SD = 0.9). Cognitive tests completed were the NART [61], Raven's Standard Progressive Matrices [58], Rey Auditory Verbal Learning Test (AVLT) [59], Digit Symbol and Block Design sub-tests of the Wechsler Adult Intelligence Scale-Revised [63], and the Uses of Common Objects Test [59].

### Manchester and Newcastle Longitudinal Studies of Cognitive Ageing Cohorts

The University of Manchester Age and Cognitive Performance Research Centre (ACPRC) programme began in 1983 and has documented longitudinal trajectories in cognitive function in 6371 older adults in the North of England. The group comprises 1917 men and 4454 women with mean age 65.6 years (SD = 14.3) at initial recruitment. Details of the battery of cognitive function tests used in alternating batteries can be found in Rabbitt *et al.* [64]. The Dyne Steel DNA Archive for Ageing and Cognition was established following invitation to all participating volunteers 1999 and 2004. This resulted in 1829 volunteers attending Manchester or Newcastle Universities, or being visited at home for blood sample collection.

### Construction of Cognitive Phenotypes

We constructed cognitive phenotypes of fluid- and crystallized-type intelligence for each of the cohorts. To represent crystallized intelligence (*g_c_*), we used the NART in the Lothian Birth Cohorts of 1921 and 1936, and the Aberdeen Birth Cohort of 1936, and the Mill Hill Vocabulary A and B vocabulary tests in the Manchester and Newcastle cohorts. All are vocabulary-based tests and are good representatives of the underlying construct of crystallized intelligence. The fact that not all cohorts received precisely the same vocabulary test introduces a phenotypic heterogeneity that is only likely to slightly reduce the size of any observed association between CNV indices and intelligence.

A general intelligence factor for fluid-type intelligence was derived in the Scottish cohorts using principal components analyses (PCA), with higher values of the components reflecting better ability. Strictly speaking, PCA does not produce ‘factors’, but this is a common usage. For the two Lothian Birth Cohorts, and the Aberdeen Birth Cohort of 1936, the scores on a number of fluid-type intelligence tests were subjected to PCA. In all cases, inspection of the scree slope and the Eigenvalues-greater-than-one criterion indicated a single component that was then extracted. Individuals' scores on the first unrotated principal component were used to represent fluid-type general intelligence (*g_f_*). In LBC1921, the tests used to construct *g_f_* were the Moray House Test, Raven's Matrices, Logical Memory, and Verbal Fluency. In LBC1936, the six tests from the WAIS-III^UK^ described above were used to construct *g_f_*. The tests used to define *g_f_* in ABC1936 were Raven's Progressive Matrices, Digit Symbol, Uses of Common Objects, and AVLT. The first principal component accounted for 49% of variance in ABC1936, 56% in LBC1921 and 51% in LBC1936. The range of tests administered to the LBC1936 sample allowed the construction of the same *g_f_* battery used in LBC1921 using the LBC1936 data, and the correlation between the g_f_ scores derived from two different sets of tests on LBC1936 was ∼0.7. For the Manchester and Newcastle cohorts, a general fluid-type intelligence ability factor, *g_f,_* was obtained from a random effects model fitted by maximum likelihood to the standardized age regressed residuals obtained for each sex from tests including the Alice Heim 4 (AH4) parts 1 and 2 general intelligence tests. Detailed task descriptions can be found in Rabbit *et* *al*. [63]. The *g_f_* scores were derived separately for males and females in the Manchester and Newcastle cohorts. Although different tests were used to construct the general fluid intelligence factors between cohorts, the correlation between such factors when they are derived on the same sample tends to be very high [5], [65]. All phenotypes described above were corrected for age, and for sex for those not derived separately by gender. Standardized residuals were used in all subsequent analyses.

### SNP Genotyping and Quality Control

Genomic DNA was isolated using standard procedures at the Wellcome Trust Clinical Research Facility (WTCRF) Genetics Core, Western General Hospital, Edinburgh for LBC1936 and ABC1936, and Medical Research Council Technology, Western General Hospital, Edinburgh for LBC1921. The UK DNA Banking Network was used for the Manchester and Newcastle Dyne-Steele samples. In total, 3782 samples were genotyped at the WTCRF Genetics Core (http://www.wtcrf.ed.ac.uk) using the Illumina610-Quadv1 chip (LBC1936 N = 1,042; LBC1921 N = 526; ABC1936 N = 456; Manchester N = 901; and Newcastle N = 877).

Samples were subjected to the following quality control (QC) procedures: individuals where self-reported gender disagreed with genetic evidence were removed. Pairwise IBD between individuals was estimated and, where it was greater than 0.25, one of each pair was removed from the analysis. Samples with SNP call rate <0.95, and those showing evidence of non-Caucasian origin by multi-dimensional scaling were also removed. After QC, a total of 3,511 samples remained (LBC1936 N = 1,005; LBC1921 N = 517; ABC1936 N = 426; Manchester N = 805; and Newcastle N = 758). SNPs were retained for analyses that met the following criteria: call rate ≥0.98, minor allele frequency ≥0.01, and Hardy-Weinberg Equilibrium test with P≥0.001. A total of 549,754 SNPs passed these QC criteria and, with the inclusion of a further 21,890 non-polymorphic markers in known CNV regions, 571,644 markers in total were used to call CNVs.

### CNV Calling and Quality Control

CNV calling used Log-R-Ratio (LRR) and B-allele Frequency (BAF) values normalised and extracted from raw signal data using Illumina's Genome Studio software. CNV calling was performed using the detect_cnv.pl script from PennCNV [66], and the QuantiSNP package [67]. Only variants that were called by both algorithms were used in the analysis. Where CNV boundaries were not identical between the two algorithms, the start and end of the overlapping region were taken as the CNV boundaries. Quality control steps [54] were applied at the level of sample quality, and of individual CNVs. Twenty samples were genotyped in duplicate to check consistency of genotype calling. Before QC steps were implemented, 47% of total CNVs called were common to both samples, increasing to 67% as restrictions on CNV length (≥ 500kb) were put in place. After QC, the sample in each pair with the lower SNP call rate was excluded from further analysis. To investigate the effect of rare CNVs, the QC and selection procedures of Williams *et al.* [54] were followed. Briefly, samples with standard deviation of Log-R Ratio across all markers greater than 0.3 were excluded, as were samples with 30 or more CNVs called at longer than 100kb, due to the unreliability of these calls. Quality control was also performed at the level of individual CNVs, with variants that spanned fewer than 15 contiguous markers discarded. Any adjacent CNVs that appeared to be artificially separated by the calling algorithm were merged: CNVs were candidates for merging when pairs of adjacent variants on the same chromosome, of the same copy number state, were greater than 200kb in length and separated by a distance of less than half the total length of the merged variant. LRR and BAF data for all candidate merges were inspected visually. To investigate the effect of only rare variants, CNVs present in greater than 1% of each cohort were discarded from analysis. CNV boundaries do not necessarily correspond exactly between samples, so variants were removed where any marker along their length was called in a CNV region in greater than 1% of each cohort. We investigated the effect of removing common CNVs using the 1% criterion on the entire sample, rather than individual cohorts, and found no differences in the significance of results between the two sets of data (not shown).

### Modelling CNV load

To investigate the effect of CNV load, we derived three variables for each individual: the total number of CNVs that passed the QC criteria outlined above; the total length of these variants; and the number of genes disrupted by these CNVs. Genes were counted as ‘disrupted’ if there was any overlap between called CNV regions and known genetic co-ordinates +/−20 kb. The effect of CNV load on intelligence was investigated by fitting linear regression models to derived intelligence (*g*) factors and test scores. A number of regression models were fitted, using residualised *g_f_* and *g_c_* factor scores, corrected for age and sex effects, against total number of CNVs (rate), total CNV length, and the total number of genes disrupted, with ‘cohort’ fitted as a covariate.

### CNV regions

Numerous copy-number variable regions have been implicated in mental health disorders. Williams *et al.* [54] identify 20 regions that have been associated with schizophrenia or autism spectrum disorders. We investigated the effect of these specific variants in our cohorts; i.e., we checked whether any individuals carried the disease-associated variants reported in autism spectrum disorders [45] or schizophrenia [51]. Where more than two individuals within the sample carried one of these variants, the differences in means of the carriers and non-carriers were tested using a *t*-test in R. Permutation analysis was performed to generate corrected p-values, on 100,000 permutations of the data. For each permutation, phenotypic values were permuted with reference to individual IDs, and a *t*-test was performed for each CNV region identified in Williams *et al.* [54]. The maximum test statistic of these 20 tests was retained at each permutation. Observed p-values were compared to the distribution of test statistics, with empirical p-values calculated for each region as the proportion of permutations where the maximum test statistic was greater than the observed statistic.

## Results

Following the QC procedures outlined above, 3133 individuals remained with phenotypic information for *g_f_*, and 3210 for *g_c_.* The CNVs used in subsequent analyses were those that fulfilled the QC procedures outlined above, and were called as CNVs by both QuantiSNP and PennCNV. For the samples providing the cognitive phenotypes *g_f_*, and *g_c_*, the total numbers of long, rare CNVs at ≥500 kb present in ≤1% of the cohort samples was 167 for both phenotypes. This gives overall CNV rates of 0.053 and 0.052 CNVs per individual for *g_f_* and *g_c_* (tables 1 and 2), with the slight discrepancy in total CNV counts due to different numbers of individuals with each phenotype. Most individuals carried no rare CNVs and, of those carrying any, the majority carried a single variant, with only nine individuals carrying more than one (Tables 3 and 4). Variants are observed across all autosomes throughout the sample, with more observed on longer chromosomes. The numbers of genes disrupted by these CNVs are shown in Tables 5 and 6 for *g_f_* and *g_c_* respectively, determined by comparing CNV boundaries to genetic start and end co-ordinates (+/−20 kb) and counting the number that overlap.

**Table 1:** Total CNV burden in each cohort for fluid-type intelligence (*g_f_*).

		All CNVs		Deletions		Duplications	
Cohort	Sample Size	Load	Rate	Load	Rate	Load	Rate
ABC1936	346	12	0.035	2	0.006	10	0.029
LBC1921	482	24	0.050	8	0.017	16	0.033
LBC1936	877	47	0.054	15	0.017	32	0.037
Manchester	730	44	0.060	12	0.017	32	0.044
Newcastle	698	40	0.057	4	0.006	36	0.052
Total N	3133	167	0.053	41	0.013	126	0.040

Total *N* represents the number of individuals with *g_f_* phenotypes, and high quality genetic data used to call CNVs. Load is the total number of CNVs counted in each cohort, called by both PennCNV and QuantiSNP, that passed quality control criteria, namely longer that 500 kb, and present at a frequency of 1% or less within each cohort, and Rate is the average number of such CNVs per individual within each cohort, with totals for All CNVs called, and separated into Deletions and Duplications.

**Table 2:** Total CNV burden in each cohort for crystallized-type intelligence (*g_c_*).

		All CNVs		Deletions		Duplications	
Cohort	Sample size	Load	Rate	Load	Rate	Rate	Load
ABC1936	412	12	0.029	2	0.005	10	0.024
LBC1921	492	24	0.049	8	0.016	16	0.033
LBC1936	887	47	0.053	15	0.017	32	0.036
Manchester	723	44	0.061	12	0.017	32	0.044
Newcastle	696	40	0.058	4	0.006	36	0.052
Total *N*	3210	167	0.052	41	0.013	126	0.039

Total *N* represents the number of individuals with *g_c_* phenotypes, and high quality genetic data used to call CNVs. Load is the total number of CNVs counted in each cohort, called by both PennCNV and QuantiSNP, that passed quality control criteria, namely longer that 500 kb, and present at a frequency of 1% or less within each cohort, and Rate is the average number of such CNVs per individual within each cohort, with totals for All CNVs called, and separated into Deletions and Duplications.

**Table 3:** Distribution of long, rare CNVs in each cohort for individuals with a fluid-type intelligence (*g_f_*).

# Rare CNVs	ABC1936	LBC1921	LBC1936	Manchester	Newcastle	Total
0	334	458	830	686	658	2966
1	12	22	41	36	37	153
2	0	1	3	4	0	8
3	0	0	0	0	1	1
Total	346	482	877	730	698	3133

Total numbers of individuals with *g_f_* phenotype carrying 0–3 long (≥500 kb), rare (< 1% frequency) copy number variants in each cohort.

**Table 4:** Distribution of long, rare CNVs in each cohort for individuals with a crytallized-type intelligence (*g_c_*).

# Rare CNVs	ABC1936	LBC1921	LBC1936	Manchester	Newcastle	Total
0	400	468	840	679	656	3043
1	12	22	41	36	37	148
2	0	1	3	4	0	8
3	0	0	0	0	1	1
Total	412	492	887	723	696	3210

Total numbers of individuals with *g_c_* phenotype carrying 0–3 long (≥500 kb), rare (<1% frequency) copy number variants in each cohort.

**Table 5:** Total genes disrupted by CNVs in each cohort for fluid-type intelligence (*g_f_*).

		All CNVs		Deletions		Duplications	
Cohort	Sample Size	Gene Load	Rate	Gene Load	Rate	Gene Load	Rate
ABC1936	346	37	0.107	8	0.023	29	0.084
LBC1921	482	60	0.125	10	0.021	50	0.104
LBC1936	877	144	0.164	56	0.064	88	0.100
Manchester	730	81	0.111	19	0.026	62	0.085
Newcastle	698	120	0.172	4	0.006	116	0.166
Total	3133	442	0.141	97	0.031	345	0.110

Gene load is calculated as the number of genes whose co-ordinates +/−20 kb intersect with a CNV that passes QC checks (length ≥500 kb, and frequency ≤1%). Rate is the average number of such CNVs per individual.

**Table 6:** Total genes disrupted by CNVs in each cohort for crystallized-type intelligence (*g_c_*).

		All CNVs		Deletions		Duplications	
Cohort	Sample Size	Gene Load	Rate	Gene Load	Rate	Gene Load	Rate
ABC1936	412	37	0.090	8	0.019	29	0.070
LBC1921	492	60	0.122	10	0.020	50	0.102
LBC1936	887	144	0.162	56	0.063	88	0.099
Manchester	723	81	0.112	19	0.026	62	0.086
Newcastle	698	120	0.172	4	0.006	116	0.167
Total	3210	442	0.138	97	0.030	345	0.108

Gene load, is calculated as the number of genes whose co-ordinates +/−20 kb intersect with a CNV that passes QC checks (length ≥500 kb, and frequency ≤1%). Rate is the average number of such CNVs per individual.

A linear regression model was fitted on total CNV rate (number of CNVs called) per individual, fitting cohort as a covariate, summarised in Tables 7 and 8 for *g_f_* and *g_c_* respectively,. Results are also shown for similar regressions performed on the total length of CNVs per individual, and for the number of genes disrupted by CNVs. Models were fitted using data from all types of CNV, and for deletions and duplications separately. None of the fitted regression models were significant at p<0.05 for CNV effects. For numbers of rare CNVs, we grouped individuals into “carriers” and “non-carriers” and compared the *g* scores in these two groups. We found no significant differences between them, for all CNVs, or for deletions or duplications alone (data not shown).

**Table 7:** Tests of significance of CNV load on regression on fluid-type (*g_f_*) intelligence.

	All			Dels			Dups		
	Effect	p-val	Emp p-val	Effect	p-val	Emp p-val	Effect	p-val	Emp p-val
CNV count	−0.002	0.927	0.913	−0.010	0.592	0.606	+0.004	0.826	0.822
CNV length	−0.014	0.419	0.425	−0.018	0.326	0.334	-0.007	0.712	0.719
Genes disrupted	−0.020	0.261	0.283	−0.035	0.053	0.055	-0.004	0.802	0.822

Effect sizes are reported as standardized β values for each regression model, fitting total CNV count, length and number of genes disrupted against fluid-type (*g_f_*) intelligence for rare CNVs of length ≥500 kb present at ≤1% frequency in each cohort. Regression models fitted for all CNVS (all), deletions only (Dels) and duplications only (Dups). P-values for regression tests are given for each regression, along with empirical p-values, calculated from 100,000 permutations of each model.

**Table 8:** Tests of significance of CNV load on regression on crystallized-type (*g_c_*) intelligence.

	All			Dels			Dups		
	Effect	p-val	Emp p-val	Effect	p-val	Emp p-val	Effect	p-val	Emp p-val
CNV count	−0.012	0.513	0.502	−0.006	0.743	0.743	-0.010	0.567	0.592
CNV length	−0.005	0.774	0.752	−0.007	0.703	0.687	-0.002	0.910	0.907
Genes disrupted	−0.010	0.555	0.553	−0.025	0.150	0.149	+0.002	0.926	0.938

Effect sizes are reported as standardized β values for each regression model, fitting total CNV count, length and number of genes disrupted against crystallized-type (*g_c_*) intelligence for rare CNVs of length ≥500 kb present at ≤1% frequency in each cohort. Regression models fitted for all CNVS (all), deletions only (Dels) and duplications only (Dups). P-values for regression tests are given for each regression, along with empirical p-values, calculated from 100,000 permutations of each model.

We also examined the effect of shorter, rare CNVs, at lengths 100–200 kb and 200–500 kb. These data are presented in Tables S1 and S2.Of these tests, the majority showed no association with either fluid or crystallized intelligence, but one test, total CNV counts for deletions in the range 200–500 kb, showed a nominally significant p-value of 0.039 with *g*_f_, although this is not robust to multiple testing correction. Yeo *et al.* [52] reported a significant association between rare deletions and Full-Scale IQ, but their definition of ‘rare’ (5%) differs from our 1% threshold, and other QC criteria differ. Repeating the analysis using the QC criteria of Yeo *et al.* on our samples failed to replicate their association (Tables S3 & S4).

Previous studies that have investigated the effect of rare CNVs on so-called neurocognitive disorders found several copy number variants that have an effect on these traits [39], [43], [51], [68]–[74]. Because these disorders also involve cognitive deficits, we examined variants declared significant in studies for autism and schizophrenia in our samples. The results are listed in Table S5. Where more than two individuals carried any particular variant, the sample was split into carriers and non-carriers, and differences in intelligence between these two groups were assessed using *t*-tests. Of the twenty loci examined, one region, 16p13.11, had four CNV carriers within our sample, and a region overlapping *SHANK3* had three. The 16p13.11 region showed no evidence of an effect on either *g_f_* or *g_c_*, but *SHANK3* showed a nominally significant effect at p = 0.006 for fluid-type intelligence. Permutation analyses were performed to generate corrected p-values: −100,000 permutations were performed, the largest test statistic taken over all 20 CNV regions, and the observed test statistic for each region compared to this distribution to calculate an empirical p-value. Following this procedure, *SHANK3* remained significant for *g_f_* with a corrected p-value of 0.01.(Table S5).

## Discussion

Intelligence differences are substantially heritable, but studies to date have failed to find replicable associations between SNPs and cognitive traits that account for variation in intelligence in the normal population [8], [11]. One potential source of the missing heritability is copy-number variation. Following a similar approach to Williams *et* *al.* [54], in which the combined effect of rare CNVs on variation in ADHD was investigated in a sample of 366 cases and 1047 controls, we examined whether variation in rare CNVs had any effect within older cohorts with intelligence distributions in the normal range had any effect on the variation in cognitive ability. No significant combined effect of rare CNVs was found on intelligence in our combined sample of over 3,000 elderly individuals.

We found that the total load of CNVs longer than 500 kb per individual was not significantly associated with fluid- or crystallized-type intelligence phenotypes. Neither was total length of copy number variants, nor the total number of genes disrupted by rare CNVs. Testing for differences in intelligence between individuals carrying CNVs known to effect neurocognitive phenotypes, and non-carriers found a suggestive effect of *SHANK3* on fluid intelligence scores. *SHANK3* is a post-synaptic density protein involved in the regulation of synaptic transmission, and has been implicated in both autism and schizophrenia. *SHANK3* is within the region of the chromosome 22q13.3 deletion syndrome, which is characterized by neonatal hypotonia, global developmental delay, severe cognitive deficits, normal to accelerated growth, absent to severely delayed speech, autistic behaviour, and minor dysmorphic features [73], [75]. Haploinsufficiency of this gene as a major causative factor in the neurologic symptoms of 22q13 deletion syndrome [76], and Gauthier et al. [77] identified two de novo mutations (R1117X and R536W) in two families with schizophrenia, in patients also displaying borderline or mild mental retardation. A recent GWAS study on cognitive phenotypes which used SNPs to tag common CNVs [78], found no significant association between these tagging markers and any of the measured cognitive phenotypes after correcting for multiple testing. This study by Need *et al.* [78] focussed on associations between cognitive phenotypes and specific copy-number variants associated with psychiatric illnesses in a sample of 1,000. Similarly, Saus *et al.* [79], using a candidate gene approach, found no significant differences between rare variants in cases and controls in major depressive disorder, bipolar disorder, schizophrenia or anxiety disorders, testing a control group of 341 individuals against case samples of ∼200 per disorder.

Comparing our results to Yeo *et al.* [52], who found significant associations between rare deletions and variation in intelligence in a sample of 77 individuals, we failed to replicate their observed significant association between the extent of rare deletions and general intelligence, even when applying the same set of CNV QC criteria to our larger data set There are several possible reasons why this may be so. Whereas the phenotypic measures of intelligence that were used are broadly comparable, there are slight differences in our CNV calling and QC methods, the major one being our use of a minimum 500 kb cut-off in CNV length, but no such cut-off was used by Yeo *et al.* This is apparent from the overall rate of CNVs per individual in the two studies, with Yeo *et al.* finding an average of 17.42 CNVs per individual, with an average deletion rate of 10.95 (SD = 5.48) and insertion rate of 6.47 (SD = 9.82); compared to our observations of ∼0.05 in total, a substantial disparity between the two rates, and between Yeo *et al.* and other studies of ‘rare’ CNVs which include a length cut-off [80]. The rationale behind such a cut-off is that the calls for longer CNVs are more accurate than shorter variants, and including short variants in an association analysis may increase the probability of type-I errors. When applying the QC criteria of Yeo *et al.* to our data (removing the length restriction, and excluding common CNVs at the 5% threshold), the observed average rate of CNVs called per individual was 15.48 (SD = 7.59). None of the regression analyses performed on CNVs called using these criteria showed a significant effect in our sample (Tables S3 and S4).

The samples studied in the present report comprise individuals within the normal cognitive range and we would expect the rate of CNVs detected to be similar to that obtained in other groups of healthy individuals that have undergone the same CNV quality control and selection procedures. Several studies have investigated the effect of long, rare CNVs on disease susceptibility, using the same length and frequency criteria employed here. Comparing our rates of CNV detection to the control groups of these studies, our observed values of 0.052 and 0.053 are comparable to the values of 0.05 observed by Blauw *et al.* [49], slightly lower than the value of 0.075 reported by Williams *et al.* [54], and lower than the values of 0.12 reported by the ISC [51], 0.17 in Pinto *et* *al.* [50], and 0.1924 in Bochukova *et al.* [81]. Some of these discrepancies can partially be accounted for by differences in genotype platforms and CNV calling algorithms. Blauw *et al.* [49] genotyped their control samples using a number of different platforms, but analysed only CNVs called by markers common to all: these were effectively the markers present on the HumanHap 300 array, comprising ∼300 K SNP markers and lacking CNV specific probes. The ISC study [51] used genotypes derived from Affymetrix 5.0 and 6.0 arrays, and noted differences between these two arrays within their study. Bochukova *et al.* [81] also used the Affymetrix 6.0, whereas Pinto *et al.* [50] used the Illumina 1M chip for genotyping, and both observed a higher rate of CNVs per sample. Whenever a minimum number of SNPs is used as a criterion to define a CNV, there will be *de facto* more CNVs called on chips with higher marker densities.

Although the absolute rates of CNVs called differ between studies, the proportions of deletions detected compared to duplications are more consistent between studies. Of our total observed variants, 24.6% (41/167),are deletions in, compared to 28.0% in Bochukova *et al.* [81], 28.3% in ISC, and 29.2 % in Pinto *et al* [50]. The anomalously large proportion of 72.0% deletions in Blauw *et al.* [82] may be due to the set of markers used detecting more deletions. The 16.7% duplications called in Williams *et al.* [54] is smaller than other studies, perhaps due to the small sample size.

There are several other reasons why we may have failed to detect any effect of rare CNVs on cognitive ability in the cohorts analysed here. Primary amongst them, we are unlikely to have captured all of the genetic variation present within our samples. The Illumina 610 Quad chip used for genotyping in this study contains several non-polymorphic markers in known copy number variable regions, but will still not capture all of the variation present. With subsequent developments in microarrays, such as the Illumina 1M array used by Yeo *et al.*, and the advent of reasonably priced whole-genome sequencing, we could capture a higher proportion of the actual variation. Other factors beyond genetic and structural variation may also contribute towards variation in general cognitive ability, including environmental variation and gene methylation or other epigenetic effects. Significant regions for rare CNVs reported in other neurocognitive disorders are not found significant by genome-wide associations using SNPs. Need *et al.* [78] suggest that looking for the effect of rare variants enriched in schizophrenia patients within a healthy population may reveal an association. Of the regions we examined, *SHANK3* remained significant following permutation analysis, suggesting that this gene may be involved in normal variation in fluid intelligence. However, of the three CNV carriers identified in our samples that overlap the *SHANK3* region, two carried duplications (copy number 3) and one a deletion (copy number 1), suggesting that imbalance rather than copy number *per se* may be important, but this counter-intuitive observation requires further investigation.

Many illnesses are associated with lower cognitive ability, and there is evidence that these states often involve structural genetic variation. Cognitive impairment is often a symptom in genomic syndromes associated with specific large-scale structural variation, including Williams-Beuren, Smith-Magenis, and Velo-Cardio-Facial Syndrome amongst others [83]. These syndromes are characterised by recurrent deletions or duplications at specific loci, which are large enough to be detected using Fluorescence In-Situ Hybridisation (FISH) or other microscopic techniques. Recent advances in microarray technology have allowed detection and characterisation of sub-microscopic structural variants, and found them to be ubiquitous throughout the genome. These copy-number variants are a major source of normal human genetic variation [84], but have also been found to be associated with complex disorders, including many neuro-psychiatric conditions, (for example, ADHD, depression, schizophrenia and autism-spectrum disorders).

To conclude, we find that, within the analytical limitations of the detection system available to us, there is no evidence for the effect of total CNV load on intelligence within the normal older population. Looking at specific CNV regions, we find evidence to suggest that copy number variation in the *SHANK3*,region where copy number variation has been previously associated with susceptibility to autism and schizophrenia, is associated with normal variation in fluid intelligence. However, our study does not preclude further contributions of CNVs at either extremes of the normal range of intelligence, or indeed on an individual by individual basis. New tools, including whole genome resequencing of individuals and their relatives with life-course measures of intelligence would be valuable in further resolving the important issue of identifying genetic contributions to individual differences in intelligence.

## Supporting Information

Table S1 **Tests of significance of CNV load on regression on fluid-type intelligence (** ***g_f_*** **).**
(DOC)

Table S2 **Tests of significance of CNV load on regression on crystallized-type intelligence (** ***g_c_*** **).**
(DOC)

Table S3 **Tests of significance of CNV load on regression on fluid-type (** ***g_f_*** **) intelligence for rare CNVs present at ≤ 5% frequency in each cohort, with no length restriction.**
(DOC)

Table S4 **Tests of significance of CNV load on regression on fluid-type (** ***g_f_*** **) intelligence for rare CNVs present at ≤ 5% frequency in each cohort, with no length restriction.**
(DOC)

Table S5 **Significance of individual CNV loci previously implicated in psychiatric disorders for fluid-type (** ***g_f_*** **) and crystallized-type (** ***g_c_*** **) intelligence.**
(DOC)
