# Supplementary material for: Genetic Copy Number Variation and General Cognitive Ability
Source: PLoS One. 2012 Dec 26;7(12):e37385. doi: 10.1371/journal.pone.0037385 (PMC3530597; doi:10.1371/journal.pone.0037385)
Supplement: Table S1 — Tests of significance of CNV load on regression on fluid-type intelligence ( gf ). (DOC) [file pone.0037385.s001.doc]

**Table S1.** Tests of significance of CNV load on regression on fluid-type intelligence (*g_f_*)

|  | | Total CNVs | | | | Total CNV Length | | | | Genes disrupted | | | |
| --- | --- | --- | --- | --- | --- | --- | --- | --- | --- | --- | --- | --- | --- |
|  |  | CNV count | rate | effect | p-val | total (Mb) | rate | effect | p-val | total genes | rate | effect | p-val |
| All | 100-200kb | 919 | 0.293 | +0.005 | 0.777 | 127.12 | 0.041 | +0.001 | 0.953 | 1158 | 0.370 | +0.016 | 0.365 |
|  | 200-500kb | 558 | 0.178 | +0.016 | 0.372 | 168.34 | 0.054 | +0.009 | 0.627 | 847 | 0.270 | +0.015 | 0.412 |
|  | ≥500kb | 167 | 0.053 | -0.002 | 0.927 | 140.20 | 0.045 | -0.014 | 0.419 | 442 | 0.141 | -0.020 | 0.261 |
| Dels | 100-200kb | 477 | 0.152 | +0.008 | 0.645 | 65.68 | 0.021 | +0.007 | 0.715 | 516 | 0.165 | +0.021 | 0.230 |
|  | 200-500kb | 204 | 0.065 | -0.021 | 0.251 | 58.58 | 0.019 | +0.011 | 0.556 | 201 | 0.064 | +0.017 | 0.346 |
|  | ≥500kb | 41 | 0.013 | -0.01 | 0.592 | 34.16 | 0.011 | -0.018 | 0.326 | 97 | 0.031 | -0.035 | 0.053 |
| Dups | 100-200kb | 442 | 0.141 | -0.001 | 0.942 | 61.44 | 0.020 | -0.005 | 0.768 | 342 | 0.205 | -0.003 | 0.882 |
|  | 200-500kb | 354 | 0.113 | +0.004 | 0.805 | 109.76 | 0.0350 | +0.003 | 0.856 | 646 | 0.206 | +0.008 | 0.652 |
|  | ≥500kb | 126 | 0.040 | +0.004 | 0.826 | 106.04 | 0.034 | -0.007 | 0.712 | 345 | 0.110 | -0.004 | 0.802 |

Summary of the load of total CNV count, total CNV length and number of genes disrupted by CNVs in the entire sample with *g_f_* phenotypes. Effect sizes are reported as standardized β values for each regression model, corrected for age and sex, on total load, with cohort fitted as a covariate, Effect size reported as standardised β values for each model.
