# Supplementary material for: Genetic Copy Number Variation and General Cognitive Ability
Source: PLoS One. 2012 Dec 26;7(12):e37385. doi: 10.1371/journal.pone.0037385 (PMC3530597; doi:10.1371/journal.pone.0037385)
Supplement: Table S2 — Tests of significance of CNV load on regression on crystallized-type intelligence ( gc ). (DOC) [file pone.0037385.s002.doc]

**Table S2**. Tests of significance of CNV load on regression on crystallized-type intelligence (*g_c_*).

|  | | Total CNVs | | | | Total CNV Length | | | | Genes disrupted | | | |
| --- | --- | --- | --- | --- | --- | --- | --- | --- | --- | --- | --- | --- | --- |
|  |  | CNV count | rate | effect | p-val | total (Mb) | rate | effect | p-val | total genes | rate | effect | p-val |
| All | 100-200kb | 915 | 0.285 | -0.019 | 0.276 | 126.48 | 0.039 | -0.019 | 0.271 | 1119 | 0.349 | -0.022 | 0.221 |
|  | 200-500kb | 546 | 0.170 | +0.029 | 0.100 | 165.09 | 0.051 | 0.032 | 0.074 | 837 | 0.261 | +0.028 | 0.108 |
|  | ≥500kb | 167 | 0.052 | -0.012 | 0.513 | 140.20 | 0.045 | -0.005 | 0.774 | 442 | 0.138 | -0.010 | 0.555 |
| Dels | 100-200kb | 474 | 0.148 | -0.021 | 0.234 | 65.27 | 0.020 | -0.019 | 0.273 | 479 | 0.149 | -0.010 | 0.553 |
|  | 200-500kb | 196 | 0.061 | +0.036 | 0.039 | 56.48 | 0.018 | +0.033 | 0.060 | 193 | 0.060 | +0.011 | 0.527 |
|  | ≥500kb | 41 | 0.013 | -0.006 | 0.743 | 34.16 | 0.011 | -0.007 | 0.703 | 97 | 0.030 | -0.025 | 0.150 |
| Dups | 100-200kb | 441 | 0.137 | -0.006 | 0.738 | 61.22 | 0.019 | -0.08 | 0.651 | 640 | 0.200 | -0.022 | 0.215 |
|  | 200-500kb | 350 | 0.109 | +0.009 | 0.605 | 108.61 | 0.034 | +0.015 | 0.389 | 644 | 0.200 | +0.026 | 0.138 |
|  | ≥500kb | 126 | 0.039 | -0.010 | 0.567 | 106.04 | 0.033 | -0.002 | 0.910 | 345 | 0.108 | +0.002 | 0.923 |

Summary of the load of total CNV count, total CNV length and number of genes disrupted by CNVs in the entire sample with *g_c_*  phenotypes. Effect sizes are reported as standardized β values for each regression model, corrected for age and sex, on total load, with cohort fitted as a covariate, Effect size reported as standardised β values for each model.
