# Supplementary material for: Genetic Copy Number Variation and General Cognitive Ability
Source: PLoS One. 2012 Dec 26;7(12):e37385. doi: 10.1371/journal.pone.0037385 (PMC3530597; doi:10.1371/journal.pone.0037385)
Supplement: Table S3 — Tests of significance of CNV load on regression on fluid-type ( gf ) intelligence for rare CNVs present at ≤ 5% frequency in each cohort, with no length restriction. (DOC) [file pone.0037385.s003.doc]

**Table S3.** Tests of significance of CNV load on regression on fluid-type (*g_f_*) intelligence for rare CNVs present at ≤ 5% frequency in each cohort, with no length restriction.

|  | All | | Dels | | Dups | |
| --- | --- | --- | --- | --- | --- | --- |
|  | Effect | p-val | Effect | p-val | Effect | p-val |
| CNV count | -0.015 | 0.41 | -0.014 | 0.42 | -0.012 | 0.51 |
| CNV length | -0.005 | 0.79 | -0.002 | 0.92 | -0.004 | 0.80 |
| Genes Disrupted | +0.017 | 0.33 | -0.013 | 0.46 | +0.017 | 0.34 |

Effect sizes are reported as standardized β values for each regression model, fitting total CNV count, length and number of genes disrupted against fluid-type intelligence (*g_f_*) for rare CNVs present at ≤ 5% frequency in each cohort. Regression models fitted for all CNVS (all), deletions only (Dels) and duplications only (Dups).
