# Supplementary material for: Genetic Copy Number Variation and General Cognitive Ability
Source: PLoS One. 2012 Dec 26;7(12):e37385. doi: 10.1371/journal.pone.0037385 (PMC3530597; doi:10.1371/journal.pone.0037385)
Supplement: Table S4 — Tests of significance of CNV load on regression on fluid-type ( gf ) intelligence for rare CNVs present at ≤ 5% frequency in each cohort, with no length restriction. (DOC) [file pone.0037385.s004.doc]

**Table S4.** Tests of significance of CNV load on regression on crystallized -type (*g_c_*) intelligence for rare CNVs present at ≤ 5% frequency in each cohort, with no length restriction.

|  | All | | Dels | | Dups | |
| --- | --- | --- | --- | --- | --- | --- |
|  | Effect | p-val | Effect | p-val | Effect | p-val |
| CNV count | -0.018 | 0.32 | -0.016 | 0.36 | -0.019 | 0.28 |
| CNV length | -0.028 | 0.12 | -0.014 | 0.11 | -0.024 | 0.18 |
| Genes Disrupted | +0.006 | 0.74 | +0.011 | 0.55 | +0.009 | 0.60 |

Effect sizes are reported as standardized β values for each regression model, fitting total CNV count, length and number of genes disrupted against crystallized-type intelligence (*g_c_*) for rare CNVs present at ≤ 5% frequency in each cohort. Regression models fitted for all CNVS (all), deletions only (Dels) and duplications only (Dups).
