# Supplementary material for: Genetic Copy Number Variation and General Cognitive Ability
Source: PLoS One. 2012 Dec 26;7(12):e37385. doi: 10.1371/journal.pone.0037385 (PMC3530597; doi:10.1371/journal.pone.0037385)
Supplement: Table S5 — Significance of individual CNV loci previously implicated in psychiatric disorders for fluid-type ( gf ) and crystallized-type ( gc ) intelligence. (DOC) [file pone.0037385.s005.doc]

**Table S5.** Significance of individual CNV loci previously implicated in psychiatric disorders for fluid-type (*g_f_*) and crystallized-type (*g_c_*) intelligence

|  |  |  |  |  |  |  | *g_f_* | | | *g_c_* | | |  |
| --- | --- | --- | --- | --- | --- | --- | --- | --- | --- | --- | --- | --- | --- |
| Chr | Start (Mb) | End (Mb) | Locus/Gene | Dis | *N (g_f_)* | *N (g_c_)* | p-val | emp p | effect | p-val | emp p | effect | Ref(s) |
| 1 | 174.1 | 175.1 | 1q25.1 | ASD | 0 | 0 | - | - | - | - | - | - | [68] |
| 2 | 13.12 | 13.16 | 2p24.3 | ASD | 0 | 0 | - | - | - | - | - | - | [68] |
| 2 | 49.99 | 51.12 | *NRXN1* | ASD | 0 | 0 | - | - | - | - | - | - | [71] |
| 3 | 2.11 | 3.08 | *CTN4* | ASD | 1 | 1 | - | - | - | - | - | - | [74] |
| 3 | 4.37 | 4.49 | *SUMF1* | ASD | 0 | 0 | - | - | - | - | - | - | [68] |
| 3 | 122.83 | 122.87 | 3q13.33 | ASD | 0 | 0 | - | - | - | - | - | - | [68] |
| 3 | 174.59 | 175.49 | *NLGN1* | ASD | 0 | 0 | - | - | - | - | - | - | [68] |
| 4 | 144.84 | 144.85 | 4q31.21 | ASD | 0 | 0 | - | - | - | - | - | - | [68] |
| 6 | 161.68 | 163.07 | *PARK2* | ASD | 0 | 0 | - | - | - | - | - | - | [68] |
| 7 | 68.69 | 69.88 | *AUTS2* | ASD | 0 | 0 | - | - | - | - | - | - | [70] |
| 10 | 87.33 | 88.12 | *GRID1* | ASD | 0 | 0 | - | - | - | - | - | - | [68] |
| 15 | 23.12 | 23.24 | *UBE3A* | ASD | 1 | 1 | - | - | - | - | - | - | [68] |
| 16 | 29.55 | 30.08 | 16p11.2 | ASD | 0 | 0 | - | - | - | - | - | - | [43] |
| 22 | 46.44 | 49.52 | *SHANK3* | ASD | 3 | 3 | 0.006 | 0.010 | +9.15 | 0.388 | 0.391 | +1.09 | [73] |
| 1 | 144.94 | 146.29 | 1q21.1 | SCZ | 2 | 2 | - | - | - | - | - | - | [39,51] |
| 15 | 20.31 | 20.78 | 15q11.2 | SCZ | 0 | 0 | - | - | - | - | - | - | [39] |
| 15 | 28.72 | 30.3 | 15q13.2-13.3 | SCZ | 1 | 1 | - | - | - | - | - | - | [39,51] |
| 16 | 15.38 | 16.20 | 16p13.11 | SCZ | 4 | 4 | 0.903 | 0.902 | -0.13 | 0.837 | 0.836 | +0.23 | [69] |
| 16 | 29.55 | 30.08 | 16p22.1 | SCZ | 0 | 0 | - | - | - | - | - | - | [72] |
| 22 | 17.5 | 20.0 | 22q11.21 | SCZ | 0 | 0 | - | - | - | - | - | - | [39,51] |

Loci represent regions where CNVs have previously been implicated in susceptibility for schizophrenia (SCZ) or autism spectrum disorders (ASD) as indicated in the Disorder column, in the References given. START and END co-ordinates for each CNV in Megabases on the relevant chromosome. Where given, p-values refer to *t*-tests on *g_f_* or *g_c_* scores between groups of CNV carriers and non-carriers, with the number of carriers for each phenotype in the relevant column. Total sample sizes are 3133 for *g_f_* and 3210 for *g_c_*. T-tests were only performed where there were more than two carriers of CNVs at that locus. P-values that are nominally significant for p < 0.05 are shown in bold. Empirical p-values based on 100,000 permutations are shown in brackets, with t-statistics shown in square brackets for tests that were nominally significant.
